# Editorial: Manipulation of immune-vascular crosstalk in solid tumors

**DOI:** 10.3389/fimmu.2023.1295953

**Published:** 2023-10-05

**Authors:** Vijay Kumar

**Affiliations:** Department of Interdisciplinary Oncology, School of Medicine, Stanley S. Scott Cancer Center, Louisiana State University Health Science Center (LSUHSC), New Orleans, LA, United States

**Keywords:** tumor immune microenvironment, tumor immunology, solid tumor, tumor vascular networks, tumor immunity, immunotherapy, immune check inhibitor (ICI)

Advances in modern sciences such as molecular biology, genetics, and immunology have equipped us to eradicate different infectious diseases (smallpox, polio) known from ancient times. However, despite these advances, cancer is the number one killer of humans. For example, in 2023, 1,958,310 new cancer cases and 609,820 cancer deaths may occur only in the United States (1). Immune-checkpoint inhibitors (ICIs) are the latest breakthrough in cancer treatment with existing chemo and radiotherapies, which are not without side effects (2–5). Furthermore, ICIs usually benefit less than 15% of cancer patients with adverse events in a large number of patients (6). Chimeric antigen receptor (CAR) T cells (especially autologous CAR-T cells) targeting tumor-associated antigen (TAAs) has transformed the treatment of many hematological cancers (CD19 CAR T cells for leukemia) and also have a high therapeutic potential for solid tumors (7–10). Besides toxic (immune and nervous system-mediated) events associated with CAR-T cells-based immunotherapies, another hurdle in their success in treating solid cancers is resistance development and cancer cell escape in the immunosuppressive tumor microenvironment (TME) or tumor immune microenvironment (TIME) (11–13). Furthermore, allogenic CAR-T cell immunotherapy may induce severe graft-versus-host disease (GVHD) and can be easily eliminated by the immune system (9). Therefore, we have to move forward to find alternative approaches to target cancers with minimum or no side/adverse events.

Blood vascular and lymphoid systems are critical for nutrition supply and immune cell infiltration in the TME or TIME (14, 15). The immune and vascular cross-talk is also fully consistent with the seed (cancer cell) and soil (specific organ environment) hypothesis given by Stephen Paget in 1899, approximately 150 years back, to explain conditions creating an environment for metastasis (16, 17). For example, tumor metastasis depends on several factors promoting cancer cell growth, proliferation, survival, immune escape, nutrition supply, and local and distant tissue invasion. Blood vascular endothelium (VE) and lymphatic endothelium (LE) differ due to their different roles that also depend on the specific tissue environment (18–20). Of note, many cancers, such as breast cancer and melanoma, preferentially spread via lymphatics (21). Furthermore, primary tumors, like blood vasculature, also modify lymphatic vessels and draining lymph nodes (dLNs) by lymph-angiogenesis, incorporating myeloid cells into lymphatics and delivering exosome or extracellular vesicles (EVs) to dLNs for generating pretumoral niches for cancer cell metastasis (15, 22–25).

A strong connection between blood vasculature and lymphatics has emerged that can change tumor biology and immunology than previously expected (26–30). Furthermore, the cross-talk between endothelial cells, immune cells, and immune checkpoints in the TME is emerging with a therapeutic potential (31). For example, a combination of anti-angiogenic therapy and ICIs normalizes vascular-immune cross-talk to increase the antitumor immunity (32). Therefore, understanding immune-vascular cross-talk in the TME or TIME of solid cancers is critical for designing better therapies. The current Research Topic of the thematic issue with four articles focusses on this less explored area of tumor immunology and vascularization.

For example, the research article by Yang et al. in this Research Topic has explored that the efficacy of ICIs such as anti-programed cell death protein-1 (PD-1 or CD279) antibodies increases in combination with the anti-angiogenesis drug, lenvatinib (a multi-receptor tyrosine kinase inhibitor, including vascular endothelial growth factor receptor-1 (VEGFR1), VEGFR2, and VEGFR3) against hepatocellular carcinoma (HCC). The increased efficacy of the ICI with lenvatinib occurred due to the normalization of the tumor vasculature and increased antitumor immune cell infiltration in the TME/TIME. For example, at adequate doses, lenvatinib increases the integrity among endothelial cells and prevents vascular leakage. Lenvatinib treatment maintains the endothelial cell integrity by forming the neuropilin-1 (NRP-1)-platelet-derived growth factor receptor-β (PDGFR-β) complex, activating a novel cdc-2 related protein kinase 1 (Crkl)-C3G (a guanine nucleotide exchange factor for Ras-associated protein 1 or Rap1, which is a small GTPase)-Rap1 signaling pathway in endothelial cells. The details of Rap-1 signaling in cancer are discussed elsewhere (33, 34). The NRP-1/PDGFR-β complex also promotes the interaction between endothelial cells and pericytes by inducing tyrosine-phosphorylation in pericytes. Thus, normovascularization by the lenvatinib in the TME increased the antitumor activity of the ICIs to suppress HCC.

Another study in the Research Topic by Liu et al., has indicated that blocking the interaction between microRNA (miR)-27a and VE-cadherin with Blockmir CD5-2, a novel oligonucleotide-based miR-27a inhibitor inhibits angiogenesis and its combination with ICIs (anti-PD-1s) significantly reduce the HCC tumor size. CD5-2 takes care of leaky blood vessels and reduces tumor hypoxia, and ICIs increase the infiltration of antitumor immune cells in the TME or TIME. Thus, combining anti-angiogenesis approach and ICIs proves beneficial to the host to decrease the tumor burden. Therefore, different approaches combining anti-angiogenesis and ICICs are emerging to target solid cancer for developing a field called tumor vasculoimmunology, where tumor vasculature and immune environment would be studied and targeted together for lowering the tumor burden and metastasis.

Furthermore, the review article by Dianat-Moghadam et al., discusses about failure of anti-angiogenesis therapies as anticancer drugs when used alone as they completely deplete vascularization, inducing hypoxia, drug resistance, and tumor recurrence along with negatively impacting chemotherapies and immune cell infiltration in the TME/TIME. They have further discussed different challenges to target tumor vasculature and its normalization for improving cancer immunotherapies. The authors have further discussed that nanomedicine-based tumor vasculature targeting and normalization has a bright future to act as potent antitumor molecules to enhance the efficacy of existing immunotherapies such as ICIs.

I have mentioned earlier that VE or vascular endothelial cells (VECs) differ from LE or lymphatic endothelial cells (LECs) and vice versa. The fourth article in the Research Topic by Viudez-Pareja et al. discuss the immunomodulatory properties of LE in the TME/TIME. LE or LECs have unique properties to suppress or potentiate the antitumor immune response. For example, tumor-associated lymphangiogenesis can promote tumor dissemination and metastasis, as seen in breast cancers and melanoma. *Viudez-Pareza et al*, discuss the immunomodulatory properties of LE within the TME/TIME of primary tumors and tumor-dLNs. They have further discussed emerging approaches to target tumor LE or LECs to enhance antitumor immune response. Hence, understating the cross-talk between LE, VE, and immune response or cells in the TME/TIME may increase the efficacy of currently available immunotherapies, such as ICIs. Additionally, it will be interesting to study the impact of immune-vascular cross-talk targeting on cellular immunotherapies, including chimeric antigen receptor-T (CAR-T) cells. Therefore, immune-vascular cross-talk has a bright future in understanding the TIME and enhancing the efficacies of current immunotherapies.

## Author contributions

VK: Conceptualization, Writing – original draft, Writing – review & editing.
